# Intraoperative vascular Doppler ultrasound blood flow and peak systolic velocity predict early patency in hemodialysis arteriovenous fistula

**DOI:** 10.1590/1677-5449.210098

**Published:** 2022-01-07

**Authors:** Guilherme de Castro-Santos, Gabriella Yuka Shiomatsu, Rafaela Martins dos Santos Oliveira, Ricardo Jayme Procópio, Túlio Pinho Navarro

**Affiliations:** 1 Universidade Federal de Minas Gerais - UFMG, Belo Horizonte, MG, Brasil.

**Keywords:** arteriovenous fistula, patency, intraoperative ultrasound, blood flow, peak systolic velocity

## Abstract

**Background:**

Chronic kidney disease is a major public health problem. Hemodialysis is the most common renal replacement therapy. Arteriovenous fistulas (AVF) are a possible access option, but early failure rates remain high.

**Objectives:**

to investigate the value of intraoperative vascular Doppler ultrasound for predicting early AVF patency.

**Methods:**

Prospective observational study. Consecutive patients undergoing AVF were assessed with vascular Doppler ultrasonography intraoperatively and on days 1, 7, 30, and 60. Patients were divided into groups according to presence or absence of primary and secondary patency. Blood flow (BF) and peak systolic velocity (PSV) were compared. ROC curves were plotted and used to define the PSV and BF values that yielded greatest sensitivity (Sens) and specificity (Spec).

**Results:**

47 patients met the inclusion criteria and were analyzed. Higher intraoperative PSV and BF values were observed in patients who had primary and secondary patency than in patients with access failure. The values with greatest sensitivity and specificity for predicting 30-day primary patency were 106 cm/s for venous PSV (Sens: 75% and Spec: 71.4%) and 290.5 ml/min for arterial blood flow (Sens: 80.6% and Spec 85.7%). Values for 30-day secondary patency were 106 cm/s for arterial PSV (Sens: 72.7%, Spec: 100%) and 230 ml/min for venous blood flow (Sens: 86.4%, Spec100%). Values for 60-day primary patency were 106 cm/s for venous PSV (Sens: 74.4%, Spec: 62.5%) and 290.5 ml/min for arterial blood flow (Sens: 80%, Spec: 75%).

**Conclusions:**

Peak systolic velocity and blood flow measured using intraoperative vascular Doppler ultrasound can predict early patency of hemodialysis arteriovenous fistulas.

## INTRODUCTION

Chronic kidney disease (CKD) is a global public health problem.^1^ It is estimated that in 2017 there were 697.5 million cases of CKD in the global population, 16.7 million of which were in Brazil.^1^^,^^2^ The principal renal replacement therapy method is hemodialysis. It is predicted that the demand for hemodialysis will have more than doubled from 2010 to 2030.^3^

The preferred type of hemodialysis access is an autogenous arteriovenous fistula (AVF).^4^ Compared to prostheses and catheters, AVF offers better long-term patency and lower rates of complications, infections, and mortality.^5^^,^^6^ However, it is known that patency rates are not ideal and the early failure rate has a major impact, compromising around 20% of AVFs.^7^^,^^8^

Intraoperative vascular Doppler ultrasonography is a very important tool because it can be used to monitor hemodynamic variables in patients undergoing AVF creation.^9^^,^^10^ The objective of this study is to evaluate the relationship between hemodynamic variables measured with intraoperative vascular Doppler ultrasonography and early patency of hemodialysis AVFs.

## METHODS

The study was authorized by the Ethics Committee at the Universidade Federal de Minas Gerais (UFMG), Belo Horizonte, Minas Gerais (MG), Brazil, with CAAE: 03241718.6.0000.5149, decision number: 3.257.774, Brazilian Clinical Trials Registration (Rebec) number: UTN: U1111-1247-880. All patients signed free and informed consent forms, and the confidentiality of participants’ data was fully preserved throughout the process.

The design is an observational, prospective, cohort study. Patients over the age of 18 who underwent elective hemodialysis AVF creation were selected consecutively at the Hospital das Clínicas da UFMG, Belo Horizonte, MG, Brazil, from May 2019 through December 2020. Patients whose access was created using a prosthetic arteriovenous loop were excluded. In order to reduce the risk of selection bias, all patients who underwent creation of a definitive hemodialysis access during the study period were invited to take part in the study. All of the patients who were invited to take part agreed to participate.

Preoperative assessment included clinical examination and mapping with vascular Doppler ultrasound. The most distal arteriovenous anastomosis site possible was preferred, with minimum diameters of 2 mm for the donor artery and 3 mm for the recipient vein. The patient was also examined with vascular Doppler ultrasonography during the operation. All fistulas and vascular Doppler ultrasonography examinations were performed by the same vascular surgeon, following the hospital’s protocols.

### Surgical procedure

Radial-cephalic, brachial-cephalic, brachial-basilic, and ulnar-basilic fistulas were created according to the hospital’s protocols with brachial plexus block or local anesthesia and local intra-arterial and intravenous administration of heparin solution at 1:100. Brachial-basilic fistulas were created in a single intervention^11^ with superficial and anterior displacement of the vein. Brachial-brachial fistulas were created in a single operation or in two stages. Single-step surgery was performed using the technique described by Bazan and Schanzer.^12^ Two-step procedures consisted of side-to-side anastomosis of the brachial vessels and later superficialization of the most appropriate vein, according to maturation criteria.

### Assessment with vascular Doppler ultrasonography

Vascular Doppler ultrasonography was conducted using a Philips CX 50 US (Philips Medical Systems, Andover, MA, United States) with a L9-3 linear transducer (frequency: 9-3 MHz) for intraoperative examinations and a Toshiba Aplio 300 US (Toshiba Medical Systems, Tokyo, Japan) with a L9-3 linear matrix transducer (frequency: 9-3 MHz) for postoperative examinations. There was therefore no direct comparison of variables from the two different systems.

For the intraoperative examination, the values analyzed were those obtained immediately after arteriovenous anastomosis. The diameters of target arteries and veins were measured as the vertical distance between the external walls of the artery and vein, using the electronic measurement tool on the vascular Doppler ultrasonography machine. Hemodynamic parameters were measured with a sagittal scan of the target artery and vein, approximately 3 cm proximal to the anastomosis with optimization in ultrasound B-mode. The pulsed wave Doppler ultrasound mode was then activated and the gate was placed on the center of the arterial or venous lumen and its sample volume set to 3 mm. The angle of insonation (defined as the angle between the ultrasound beam and the direction of blood flow) was then adjusted and maintained at 60 degrees or less. The pulsed Doppler spectral wave form trace was then activated and its scale adjusted. Assuming that an ideal pulsed Doppler spectral wave form was obtained, it was traced automatically and the vessel hemodynamic parameters displayed and saved. These parameters include the peak systolic velocity (PSV, cm/s) and mean velocity (Vm, cm/s). The values shown are the mean of three consecutive cardiac cycles. The cross-sectional area of the vessel was calculated assuming that the artery and the vein had a circular cross-section. Blood flow (BF) was subsequently calculated as the product of Vm and cross-sectional area, using the Doppler vascular ultrasound machine’s software.

### Patency assessment

Patency was defined as presence of intravascular flow through the recipient vein and assessed using Doppler vascular ultrasound intraoperatively and on postoperative days 1, 7, 30, and 60. Primary patency was defined as a patent fistula with no need for any type of intervention. Secondary patency was defined as a patent fistula with or without intervention.

### Sample size calculation

Sample size was calculated using measures of asymmetrical sampling between groups (4:1), considering patency on day 30 vs. arterial BF, with alpha of 0.05 and beta of 0.2, and using data from Saucy et al.,^13^ in which mean 1 = 230 (194) and mean 2 = 98 (65), achieving sufficiency with 25+6 patients, the sample size for this study.

### Statistical analysis

Patients were allocated to groups according to presence or absence of primary and secondary patency on postoperative days 1, 7, 30, and 60. Individual, demographic, and hemodynamic intraoperative vascular Doppler ultrasound (PSV and arterial and venous BF) variables were compared between groups. Normality was assessed with the Shapiro-Wilk test. Comparisons between groups were made using the Welch *t* test, the Mann-Whitney test, and Fisher’s exact test, as appropriate, and patency prediction models were analyzed using receiver operating characteristic (ROC) curves. Patency rates were illustrated using Kaplan-Meier plots. All analyses were performed using Prism GraphPad 9.00 (GraphPad Software, San Diego, CA, United States) for iOS.

## RESULTS

**Figure 1:**
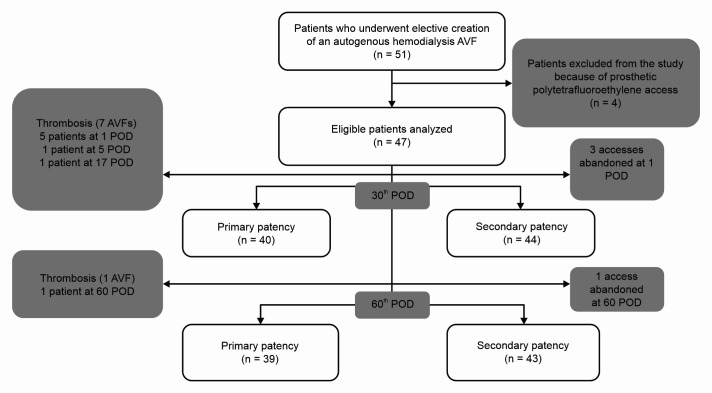
Flow diagram illustrating the study design. An abandoned access is an access that is no longer usable for dialysis. When this occurs, a new access must be created. POD = postoperative day(s).

Fifty-one patients were selected for the study. Four of them underwent creation of polytetrafluoroethylene (PTFE) prosthetic arteriovenous accesses and were excluded from the sample (Figure 1). Demographic and individual data are shown in Tables 1 and 2. No participants were lost to the sample during the period.

**Table 1:** Individual variables, comorbidities, preoperative laboratory tests, and imaging exams.

Age (mean ± SD)	56±15.8
Male (%)	22 (46)
BMI (mean ± SD)	26.4±5.9
Diabetes (%)	19 (40)
Hypertension (%)	40 (85)
Pre-dialysis (%)	33 (70)
Brachial plexus block (%)	38 (81)
Hemoglobin (mean ± SD)	11.3±1.75
Urea (mean ± SD)	120±37
Median creatinine (interquartile range)	4.43 (3.2-6.7)
Platelets – mean (minimum-maximum)	225,000 (187,000-272,000)
INR (mean ± SD)	0.96±0.05
Diameter of donor artery (mean ± SD)	3.88±1.40
Diameter of recipient vein (mean ± SD)	3.57±1.36

SD = standard deviation; BMI = body mass index; INR = international normalized ratio.

**Table 2:** Types of arteriovenous fistula and number of prior surgeries.

AVF types (%)	Radial-cephalic	17 (36.17)
	Brachial-basilic	9 (19.15)
	Brachial-brachial	2 (4.25)
	Brachial-cephalic	16 (34.04)
	Ulnar-basilic	1 (2.13)
	Ulnar-ulnar	1 (2.13)
	Radial-basilic	1 (2.13)
Previous fistulas (%)	0	66 (31)
	1	17 (8)
	2	15 (7)
	3	2 (1)

**Figure 2:**
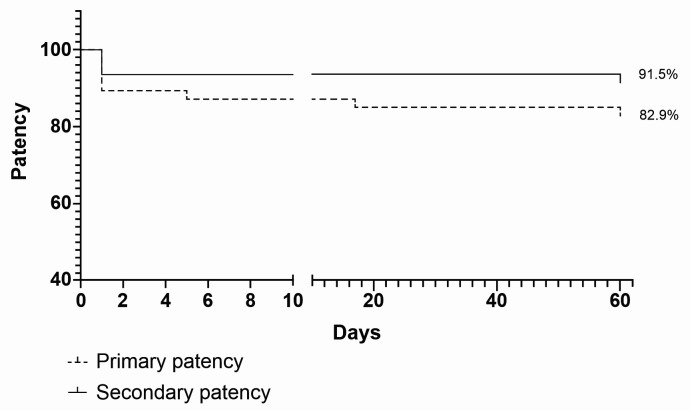
Primary and secondary patency of hemodialysis arteriovenous fistulas up to the 60th postoperative day.

Primary and secondary patency rates were, respectively, 89.4% and 93.6% on postoperative day 1; 87.2% and 93.6% on day 7; 86.96% and 96.62% on day 30; and 82.98% and 91.49% on day 60 (Figure 2). Five patients had thrombosis on day 1, two of whom underwent thromboembolectomy with a Fogarty catheter followed by reconstruction of the anastomosis and systemic anticoagulation with unfractionated heparin and warfarin; one of whom underwent creation of a more proximal substitute anastomosis; and two of whom were not reoperated. One patient suffered an occlusion on the 4th postoperative day and underwent thromboembolectomy with a Fogarty catheter followed by reconstruction of the anastomosis and systemic anticoagulation with unfractionated heparin and warfarin. This patient had two episodes of hematoma of the surgical wound, needing for surgical drainage and prompting suspension of systemic anticoagulation. On the 17th postoperative day, another patient exhibited thrombosis of the AVF, requiring thromboembolectomy with a Fogarty catheter, followed by reconstruction of the anastomosis and systemic anticoagulation with unfractionated heparin and warfarin. On the 60th postoperative day, another patient exhibited occlusion of the AVF after a puncture accident and was not reoperated (Figure 1).

**Figure 3:**
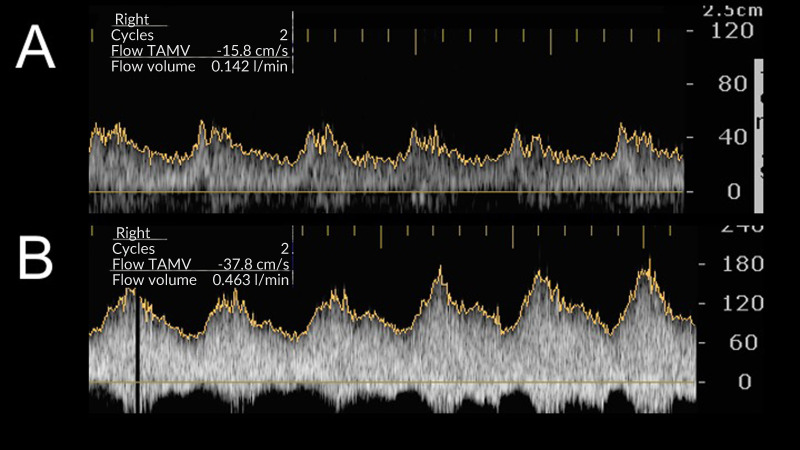
Intraoperative spectral analysis of the recipient vein soon after creation of the anastomosis. 2A) Fistula non-functional on 1st postoperative day. Peak systolic velocity (PSV) = 56 cm/s and blood flow = 142 mL/min. 2B) Fistula functional on 1st postoperative day. PSV = 192 cm/s and blood flow = 463 mL/min. TAMV = Time-average maximum velocity.

Comparison of intraoperative arterial and venous PSV and BF showed that values were higher for functional fistulas than non-functional fistulas, both when evaluated according to primary patency and according to secondary patency (Figure 3). Comparison of intraoperative arterial and venous PSV and BF between groups with and without patent AVF at 60 days revealed that values were higher for the primary patency group, but not for the secondary patency group (Table 3)

**Table 3:** Comparison of arterial and venous peak systolic velocity (PSV) and blood flow volume (BF) measured with intraoperative vascular Doppler ultrasound for patent and non-patent AVF at 30 and 60 postoperative days.

**Primary patency on 30th postoperative day. Values expressed as means and standard deviations**			
	**Yes (n = 40)**	**No (n = 7)**	**p**
PSV artery cm/s	150 (70.08)	82.93 (33.66)	0.0082
PSV vein cm/s	170 (80.72)	93.66 (46.86)	0.0038
BF artery mL/min	634 (444)	266 (261)	0.0084
BF vein mL/min	602 (462)	432 (272)	0.4451
			
**Secondary patency on 30th postoperative day. Values expressed as means and standard deviations**			
	**Yes (n = 44)**	**No (n = 3)**	**p**
PSV artery cm/s	144.4 (69.47)	72.3 (37.26)	0.0498
PSV vein cm/s	165.5 (78.8)	58.47 (37.69)	0.0104
BF artery mL/min	605.7 (439)	156.3 (100.1)	0.0132
BF vein mL/min	557.6 (330.8)	147.3 (78.14)	0.0028
			
**Primary patency on 60th postoperative day. Values expressed as means and standard deviations**			
	**Yes (n = 39)**	**No (n = 8)**	**p**
PSV artery cm/s	150.2 (70.92)	88.79 (35.30)	0.0157
PSV vein cm/s	170.3 (81.76)	102, (49.49)	0.0065
BF artery mL/min	629.5 (449.6)	333.3 (308.1)	0.042
BF vein mL/min	552.5 (323.7)	428.5 (397.9)	0.1185
			
**Secondary patency on 60th postoperative day. Values expressed as means and standard deviations**			
	**Yes (n = 43)**	**No (n = 4)**	**p**
PSV artery cm/s	144.7 (70.26)	86.68 (41.86)	0.1058
PSV vein cm/s	165.6 (79.73)	84.10 (59.8)	0.0508
BF artery mL/min	600.6 (443.8)	318.3 (334.0)	0.1411
BF vein mL/min	394.8 (498.9)	544.1 (322.3)	0.146

**Figure 4:**
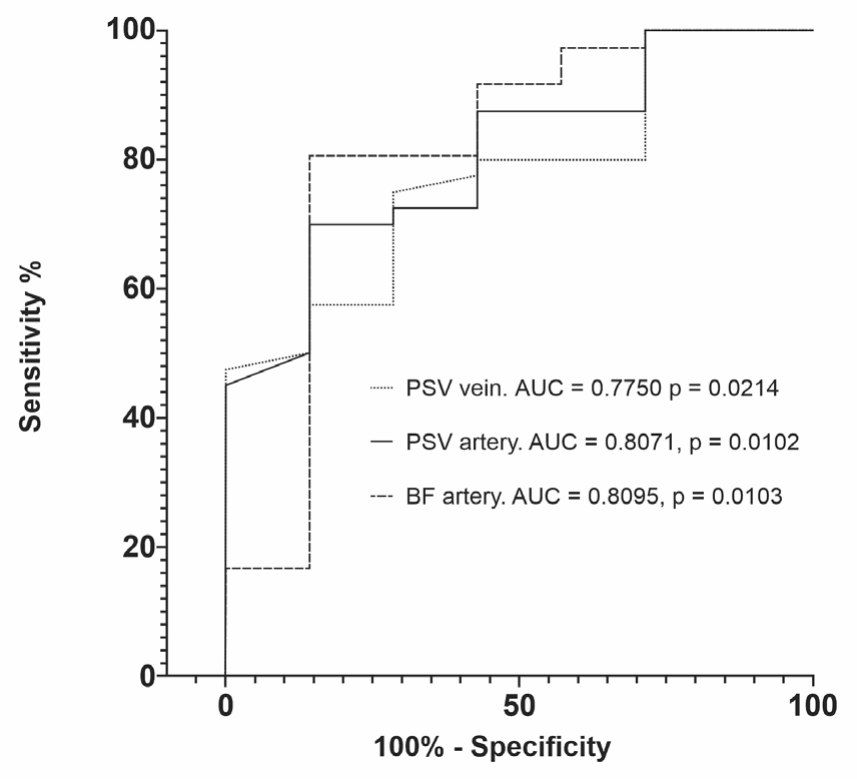
Receiver operating characteristic (ROC) curves comparing intraoperative vascular Doppler ultrasound variables and primary patency at 30 days. PSV = peak systolic velocity; BF = blood flow; AUC = area under the curve.

These results were used to construct predictive models of primary and secondary patency on the 30th day after creation of the AVF and primary patency on the 60th day, according to hemodynamic variables. Among the several ROC curves plotted for 30-day primary patency, arterial BF proved to be the variable with greatest predictive value (area under the curve [AUC] = 0.8095) (Figure 4). The cutoff points with greatest sensitivity and specificity for predicting functional AVFs with primary patency on the 30th postoperative day were 105.5 cm/s for arterial PSV, with sensitivity of 72.5% (95% confidence interval [95%CI]: 57.17-83.89%) and specificity of 71.4% (95%CI: 35.89-94.92%); 106 cm/s for venous PSV, with sensitivity of 75% (95%CI: 59.81-85.81%) and specificity of 71.4% (95%CI: 35.89-94.92%); and 290.5 mL/min for arterial BF, with sensitivity of 80.6% (95%CI: 64.97-90.25%) and specificity of 85.7% (95%CI: 48.69-99.27%) (Table 4).

**Table 4:** Sensitivity and specificity of a selection of cutoff points for arterial and venous peak systolic velocity (PSV) and blood flow (BF) measured with intraoperative vascular Doppler ultrasound for detection of primary and secondary patency at 30 postoperative days and primary patency at 60 postoperative days.

**30-day primary patency**					
	**Value**	**Sensitivity**	**95%CI**	**Specificity**	**95%CI**
PSV artery cm/s	> 99.40	72.5	57.17% to 83.89%	57.1	25.05% to 84.18%
	> 105.5	72.5	57.17% to 83.89%	71.4	35.89% to 94.92%
	> 108.5	70.0	54.57% to 81.93%	71.4	35.89% to 94.92%
PSV vein cm/s	> 93.85	80.0	65.24% to 89.50%	57.1	25.05% to 84.18%
	> 99.15	77.5	62.50% to 87.68%	57.1	25.05% to 84.18%
	> 106.0	75.0	59.81% to 85.81%	71.4	35.89% to 94.92%
BF artery mL/min	> 262.5	80.6	64.97% to 90.25%	71.4	35.89% to 94.92%
	> 290.5	80.6	64.97% to 90.25%	85.7	48.69% to 99.27%
	> 318.5	77.8	61.92% to 88.28%	85.7	48.69% to 99.27%
					
**30-day secondary patency**					
	**Value**	**Sensitivity**	**95%CI**	**Specificity**	**95%CI**
PSV artery cm/s	> 93.85	77.3	63.01% to 87.16%	66.7	11.85% to 98.29%
	> 99.15	75.0	60.56% to 85.43%	66.7	11.85% to 98.29%
	> 106.0	72.7	58.15% to 83.65%	100.0	43.85% to 100.0%
BF vein mL/min	> 219.0	88.6	76.02% to 95.05%	66.7	11.85% to 98.29%
	> 225.0	86.4	73.29% to 93.60%	66.7	11.85% to 98.29%
	> 230.0	86.4	73.29% to 93.60%	100.0	43.85% to 100.0%
BF artery cm/s	> 238.5	80.0	65.24% to 89.50%	66.7	11.85% to 98.29%
	> 251.5	77.5	62.50% to 87.68%	66.7	11.85% to 98.29%
	> 262.5	77.5	62.50% to 87.68%	100.0	43.85% to 100.0%
					
**60-day primary patency**					
	**Value**	**Sensitivity**	**95%CI**	**Specificity**	**95%CI**
PSV artery cm/s	> 99.40	71.8	56.22% to 83.46%	50.0	21.52% to 78.48%
	> 105.5	71.8	56.22% to 83.46%	62.5	30.57% to 86.32%
	> 108.5	69.2	53.58% to 81.43%	62.5	30.57% to 86.32%
PSV vein cm/s	> 106.0	74.4	58.92% to 85.43%	62.5	30.57% to 86.32%
	> 111.1	71.8	56.22% to 83.46%	62.5	30.57% to 86.32%
	> 114.6	69.2	53.58% to 81.43%	62.5	30.57% to 86.32%
BF artery mL/min	> 262.5	80.0	64.11% to 89.96%	62.5	30.57% to 86.32%
	> 290.5	80.0	64.11% to 89.96%	75.0	40.93% to 95.56%
	> 318.5	77.1	60.98% to 87.93%	75.0	40.93% to 95.56%

95%CI = 95% confidence interval.

**Figure 5:**
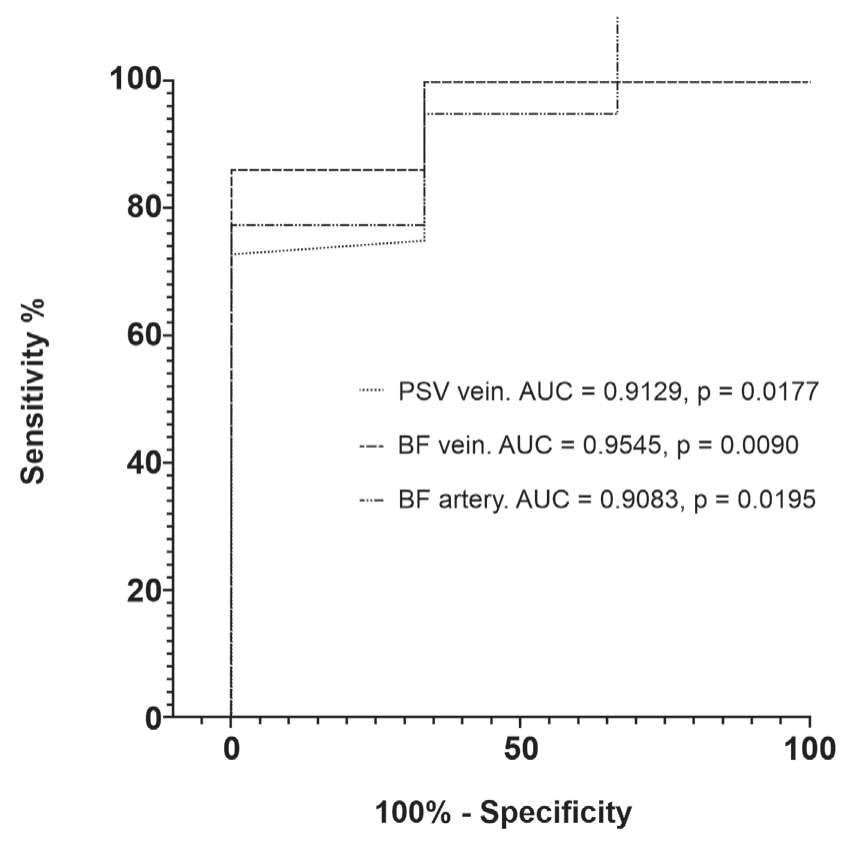
Receiver operating characteristic (ROC) curves comparing intraoperative vascular Doppler ultrasound variables and secondary patency at 30 days. PSV = peak systolic velocity; BF = blood flow; AUC = area under the curve.

The variable with greatest predictive value for secondary patency 30 days after creation of the AVF was venous BF (AUC = 0.9545) (Figure 5). The cutoff points with greatest sensitivity and specificity to predict this outcome were 106 cm/s for arterial PSV, with sensitivity of 72.7% (95%CI: 58.15-83.65%) and specificity of 100% (95%CI: 43.85-100%); 230 mL/min for venous BF, with sensitivity of 86.4% (95%CI: 73.29-93.60%) and specificity of 100% (95%CI: 43.85-100%); and 262.5 mL/min for arterial BF, with sensitivity of 77.5% (95%CI: 62.5-87.68%) and specificity of 100% (95%CI: 43.85-100%) (Table 4).

**Figure 6:**
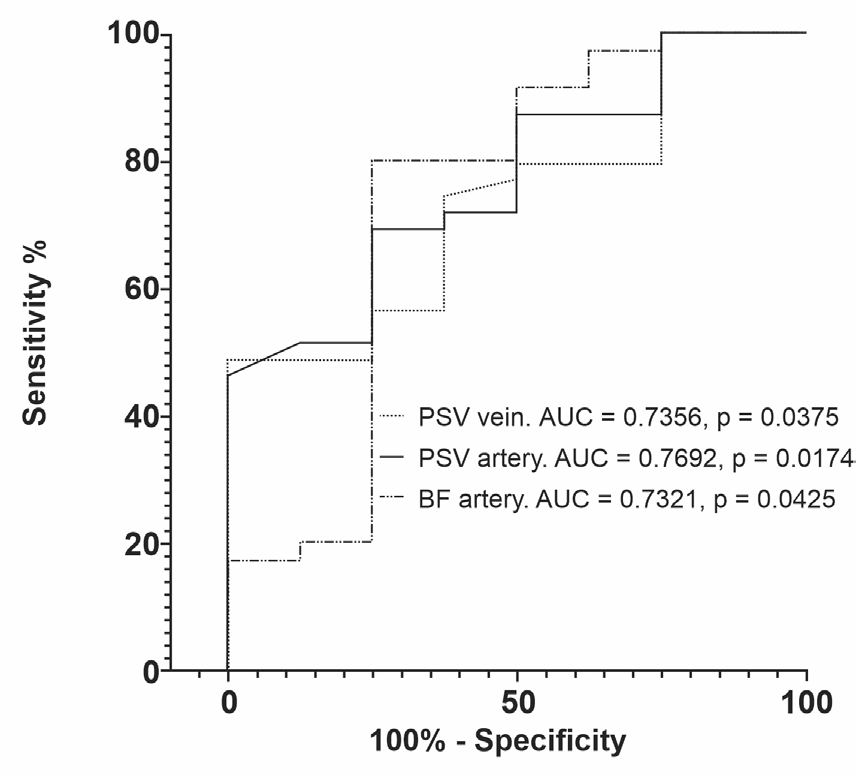
Receiver operating characteristic (ROC) curves comparing intraoperative vascular Doppler ultrasound variables and primary patency at 60 days. PSV = peak systolic velocity; BF = blood flow.

For primary patency on the 60th postoperative day, arterial PSV, venous PSV, and arterial BF had AUC of 0.7692, 0.7356, and 0.7321, respectively (Figure 6). The cutoff points yielding the greatest values for sensitivity and specificity for this outcome were 105.5 cm/s for arterial PSV, with sensitivity of 71.8% (95%CI: 56.22-83.46%) and specificity of 62.5% (95%CI: 30.57-86.32%), 106 cm/s for venous PSV, with sensitivity of 74.4% (95%CI: 58.92-85.43%) and specificity of 62.5% (95%CI: 30.57-86.32%), and 290.5 mL/min for arterial BF, with sensitivity of 80% (95%CI: 64.11-89.96%) and specificity of 75% (95%CI: 40.93-95.56%).

## DISCUSSION

Intraoperative hemodynamic parameters proved to be potential predictors of hemodialysis AVF patency. Arterial BF exhibited the greatest predictive value for primary patency on the 30th day, with sensitivity exceeding 80% with a cutoff point of 290.5 mL/min. In turn, venous BF was the variable with greatest predictive value for secondary patency on the 30th day, with sensitivity exceeding 85% and specificity of 100% with a cutoff point of 230 mL/min. Arterial and venous PSV demonstrated predictive power for primary patency on the 60th day, with cutoff points of 105.5 cm/s and 106 cm/s, respectively, yielding sensitivity in excess of 70%.

Several studies have already demonstrated an association between intraoperative BF and the main outcomes of AVFs. Saucy et al.^13^ analyzed predictors of failure to mature within 30 days of creation of radial-cephalic AVFs, identifying an intraoperative BF cutoff point of 120 mL/min, but with a lower patency rate (77.58%). Cyrek et al.^14^ compared. radial-cephalic AVFs with high (> 200 mL/min) and low intraoperative BF (< 200 mL/min), observing significantly higher primary and secondary 1-year patency rates in high-flow AVFs (100% and 93.15%, respectively) when compared with low-flow AVFs (81.25% and 75%, respectively). Other observational studies have reiterated this same relationship between postoperative BF and AVF failure, identifying cutoff points ranging from 160 mL/min to 300 mL/min.^15^^,^^16^

With regard to PSV values, several studies have confirmed the association observed. Karanan et al.^17^ analyzed AVF outcomes according to the arterial PSV value on postoperative days 1 and 8, confirming that PSV was a significant predictor of AVF outcome. Similarly, Abreu et al.^18^ conducted a 5-year follow-up study that demonstrated that PSV in ulnar and radial arteries had predictive value for secondary patency.

We observe certain limitations of this study. It is a single-center study with a single observer, so interobserver variations and biases were neither assessed nor validated. The sample was highly heterogeneous, with a wide variety of AVF types. Two different ultrasonography systems were employed. A Philips CX 50 was used for intraoperative examinations and a Toshiba Aplio 300 was used for postoperative examinations. There may be a minor variation in PSV and BF values measured by the two machines. The study also had a small patient sample, so univariate analysis was employed, which can lead to certain limitations, primarily related to the possibility of predicting a given outcome. Another important limitation was the small number of primary patency failure events by day 30, which could have contributed to the very wide CIs.

The subject investigated in this study still constitutes an open question and one that has been studied little. Since intraoperative vascular Doppler ultrasonography is an investigative method that is widely available and has no side effects or risks for patients, it is an important tool for vascular surgeons. This examination provides important variables in real time, which can correlate with short-term success of the surgery and may provoke changes to the surgical strategy during the procedure, leading to re-creation of an access or even to a change of anastomosis site, depending on the PSV and BF values observed. Although PSV has lower sensitivity and specificity than BF, it is a variable that is easy to obtain and analyze and is available on almost all vascular ultrasonography systems.

## CONCLUSIONS

Peak systolic velocity and BF measured with intraoperative vascular Doppler ultrasound are predictors of early patency after AVF hemodialysis surgery. They are both reliable assessment parameters for predicting early AVF failure and offer the opportunity to change the surgical strategy intraoperatively in order to achieve better outcomes during the postoperative period.
