# Supplementary material for: Intraoperative vascular Doppler ultrasound blood flow and peak systolic velocity predict early patency in hemodialysis arteriovenous fistula
Source: J Vasc Bras. 2022 Jan 7;20:e20210098. doi: 10.1590/1677-5449.210098 (PMC8759581; doi:10.1590/1677-5449.210098)
Supplement: Translation

# Translation: O volume de fluxo e a velocidade de pico sistólico ao ultrassom vascular com Doppler intraoperatório como preditores de perviedade precoce na fístula arteriovenosa autógena para hemodiálise

**Authors:** Guilherme de Castro-Santos, Gabriella Yuka Shiomatsu, Rafaela Martins dos Santos Oliveira, Ricardo Jayme Procópio, Túlio Pinho Navarro

**Affiliations:** 1 Universidade Federal de Minas Gerais - UFMG, Belo Horizonte, MG, Brasil.

**Keywords:** fístula arteriovenosa, perviedade, ultrassom intraoperatório, volume de fluxo, velocidade sistólica de pico

## Abstract

**Contexto:**

A insuficiência renal crônica é um problema de saúde pública mundial. A hemodiálise é a principal terapia renal substitutiva. As fístulas arteriovenosas (FAV) são uma possível escolha, mas apresentam altas taxas de falência.

**Objetivos:**

Este estudo tem como objetivo avaliar a relação entre as variáveis hemodinâmicas ao ultrassom vascular com Doppler no intraoperatório e a perviedade precoce da FAV para hemodiálise.

**Métodos:**

Tratou-se de um estudo prospectivo observacional. Os pacientes consecutivos foram submetidos a FAV com ultrassonografia vascular com Doppler em intraoperatório nos dias 1, 7, 30 e 60. Eles foram divididos em grupos quanto à presença ou não de perviedade primária e secundária, e o volume de fluxo (VF) e a velocidade de pico sistólico (VPS) foram comparados. Foram realizadas curvas *receiver operating characteristic* (ROC), com definição de valores de VPS e VF com sensibilidade (S) e especificidade (E).

**Resultados:**

Foram analisados 47 pacientes, os quais preencheram os critérios de inclusão. Os valores de VPS e VF intraoperatório foram maiores nos pacientes com perviedade primária e secundária comparados àqueles com falência. Os seguintes valores apresentaram maiores sensibilidade e especificidade para predizer perviedade primária aos 30 dias: 106 cm/s para VPS venoso, S: 75%, E: 71,4%; e 290,5 mL/min para VF arterial, S: 80,6%, E: 85,7%. Para perviedade secundária aos 30 dias, foram observados: 106 cm/s para VPS arterial, S: 72,7%, E: 100%; e 230 mL/min para VF venoso, com S: 86,4%, E: 100%. Para a perviedade primária no 60º dia, foram observados: 106 cm/s para VPS venoso, S: 74,4%, E: 62,5%; e 290,5 mL/min para VF arterial, S: 80%, E: 75%.

**Conclusões:**

A velocidade de pico sistólico e o VF ao ultrassom vascular com Doppler intraoperatório são preditores de perviedade precoce na FAV para hemodiálise.

## INTRODUÇÃO

A doença renal crônica (DRC) é um problema de saúde pública mundial^1^. Em 2017, estimou-se 697,5 milhões de casos de DRC na população global, sendo 16,7 milhões apenas no Brasil^1^^,^^2^. A principal modalidade de terapia renal substitutiva é a hemodiálise. Projeta-se que a demanda pela hemodiálise aumente em mais do que o dobro entre 2010 e 2030^3^.

Entre os diferentes tipos de acesso para hemodiálise, há preferência pela fístula arteriovenosa autógena (FAV)^4^. Se comparada às próteses e aos cateteres, a FAV possui maior perviedade a longo prazo e menores taxas de complicações, infecções e mortalidade^5^^,^^6^. Entretanto, sabe-se que as taxas de perviedade não são ideais, e a falência precoce é um fator de grande impacto, atingindo cerca de 20% das FAVs^7^^,^^8^.

A ultrassonografia vascular com Doppler intraoperatória mostra-se como ferramenta de grande importância, uma vez que torna possível acompanhar variáveis hemodinâmicas nos pacientes submetidos à confecção da FAV^9^^,^^10^. Este estudo teve como objetivo avaliar a relação entre as variáveis hemodinâmicas ao ultrassom vascular com Doppler no intraoperatório e a perviedade precoce da FAV para hemodiálise.

## MÉTODOS

O estudo foi autorizado pelo comitê de ética da Universidade Federal de Minas Gerais (UFMG), Belo Horizonte, Minas Gerais (MG), Brasil, CAAE: 03241718.6.0000.5149, número do parecer: 3.257.774, registrado no Registro Brasileiro de Ensaios Clínicos (Rebec), número do UTN: U1111-1247-880. Todos os pacientes assinaram o Termo de Consentimento Livre e Esclarecido, e a confidencialidade dos dados dos participantes foi plenamente respeitada em todos os processos.

Tratou-se de um estudo observacional do tipo coorte prospectivo. Foram selecionados pacientes consecutivos, maiores de 18 anos submetidos à confecção eletiva de FAV para hemodiálise no Hospital das Clínicas da UFMG, Belo Horizonte, MG, Brasil, entre maio de 2019 e dezembro de 2020. Pacientes submetidos à confecção do acesso com prótese arteriovenosa foram excluídos. Para reduzir o risco de viés de seleção, todos os pacientes submetidos à confecção de acesso definitivo para hemodiálise no período estudado foram convidados a participar do estudo. Todos os pacientes convidados aceitaram a participação.

A avaliação pré-operatória incluiu exame clínico e mapeamento ao ultrassom vascular com Doppler. Foi dada preferência para a anastomose arteriovenosa o mais distal possível, com diâmetro mínimo para a artéria doadora de 2 mm e para a veia receptora de 3 mm. Durante o intraoperatório, foi realizado exame de ultrassonografia vascular com Doppler. As fístulas e os exames de ultrassonografia vascular com Doppler foram realizados de acordo com os protocolos do hospital e pelo mesmo cirurgião vascular.

### Procedimento cirúrgico

As fístulas radiocefálica, braquiocefálica, braquiobasílica e ulnar-basílica foram confeccionadas de acordo com os protocolos do hospital, com bloqueio do plexo braquial ou anestesia local e administração local intra-arterial e intravenosa de solução de heparina na proporção de 1:100. As fístulas braquiobasílicas foram confeccionadas em uma única intervenção^11^ com deslocamento superficial e anterior da veia. As fístulas braquiobraquiais foram realizadas em tempo único ou em dois tempos. A cirurgia em tempo único foi realizada utilizando a técnica descrita por Bazan e Schanzer^12^. A cirurgia em dois tempos foi realizada com anastomose laterolateral entre os vasos braquiais com posterior superficialização da veia mais adequada conforme os critérios de maturação.

### Avaliação com ultrassonografia vascular com Doppler

A ultrassonografia vascular com Doppler foi realizada usando os aparelhos Philips CX 50 US (Philips Medical Systems, Andover, MA, EUA) e um transdutor linear L9-3 (frequência 9-3 MHz), para os exames intraoperatórios, e Toshiba Aplio 300 US (Toshiba Medical Systems, Tóquio, Japão) e um transdutor de matriz linear L9-3 (frequência 9-3 MHz) para os exames pós-operatórios. Dessa forma, não houve comparação direta de variáveis entre aparelhos diferentes.

Para o exame intraoperatório, considerou-se os valores obtidos no momento imediatamente após a anastomose arteriovenosa. Os diâmetros da artéria e da veia-alvo foram medidos como a distância vertical entre as paredes externas da artéria e da veia, usando o paquímetro eletrônico na máquina de ultrassonografia vascular com Doppler. Os parâmetros hemodinâmicos foram realizados com uma varredura sagital da artéria e da veia-alvo, a aproximadamente 3 cm proximalmente à anastomose, e a imagem de ultrassom modo B foi otimizada. O modo de ultrassom Doppler de onda pulsada foi, então, ativado, *o gate size* foi localizado no centro do lúmen arterial ou venoso e o tamanho da amostra foi ajustado para 3 mm. Em seguida, o ângulo de insonação, definido como o ângulo entre o feixe de ultrassom e a direção do fluxo sanguíneo, foi ajustado e mantido em 60 graus ou menos. O traçado da forma de onda espectral Doppler de onda pulsada foi, então, ativado, e a escala, ajustada. Uma vez que uma forma de onda espectral Doppler de onda pulsada ideal foi alcançada, ela foi automaticamente traçada, e os parâmetros hemodinâmicos vasculares foram exibidos e registrados. Os parâmetros incluíram velocidade sistólica de pico (VPS, cm/s) e velocidade média (Vm, cm/s). Os valores exibidos eram de uma média de três ciclos cardíacos consecutivos. A área da seção transversal do vaso foi calculada assumindo que a artéria e a veia tinham uma seção transversal circular. O volume de fluxo (VF) foi subsequentemente calculado como um produto da Vm e da área transversal, utilizando o *software* da máquina de ultrassom vascular com Doppler.

### Avaliação da perviedade

A perviedade foi considerada como a presença de fluxo intravascular em veia receptora e avaliada através do ultrassom vascular com Doppler em intraoperatório e nos dias 1, 7, 30 e 60 do pós-operatório. A perviedade primária foi considerada como fístulas pérvias sem nenhum tipo de intervenção. A perviedade secundária foi considerada como fístulas pérvias com e sem intervenções.

### Cálculo do tamanho da amostra

Foram utilizadas medidas assimétricas de amostragem entre os grupos (4:1), considerando a perviedade no D30 vs. o VF da artéria, com alfa 0,05 e beta 0,2, e, utilizando os dados de Saucy et al.^13^, em que média 1 = 230 (194) e média 2 = 98 (65), haveria suficiência para 25+6 pacientes, contemplando a amostra deste estudo.

### Análise estatística

Os pacientes foram divididos em grupos quanto à presença ou não de perviedade primária e secundária nos dias 1, 7, 30 e 60 após a cirurgia. Variáveis individuais, demográficas e hemodinâmicas ao ultrassom vascular com Doppler em intraoperatório – VPS e VF arteriais e venosos – foram comparadas entre os grupos. Para avaliação da normalidade, foi utilizado o teste de Shapiro-Wilk. A comparação entre grupos foi feita por meio dos testes *t* de Welch, de Mann-Whitney e exato de Fisher, quando adequados, e os modelos de previsão de perviedade foram analisados por meio da curva *receiver operating characteristic* (ROC). As taxas de perviedade foram demonstradas por meio de curvas de Kaplan-Meier. Todas as análises foram realizadas no *software* Prism GraphPad 9.0.0 (GraphPad Software, San Diego, CA, EUA) para iOS.

## RESULTADOS

Cinquenta e um pacientes foram selecionados para o estudo. Destes, quatro foram submetidos à confecção do acesso por meio de prótese arteriovenosa de politetrafluoroetileno (PTFE) e excluídos da amostra (Figura 1). Os dados demográficos e individuais estão demonstrados nas Tabelas 1 e 2. Não houve perda de participantes no período.

**Figura 1:** Diagrama de fluxo representando o desenho do estudo. Acesso abandonado é aquele através do qual não é possível mais a realização de diálise. Quando ocorre, é necessário a confecção de um novo acesso. DPO = dias de pós-operatório.

**Tabela 1:** Variáveis individuais, comorbidades, exames pré-operatórios laboratoriais e de imagem.

Idade (média ± DP)	56±15,8
Sexo masculino (%)	22 (46)
IMC (média ± DP)	26,4±5,9
Diabetes (%)	19 (40)
Hipertensão (%)	40 (85)
Pré-dialítico (%)	33 (70)
Bloqueio de plexo braquial (%)	38 (81)
Hemoglobina (média ± DP)	11,3±1,75
Ureia (média ± DP)	120±37
Creatinina mediana (intervalo interquartil)	4,43 (3,2-6,7)
Plaquetas – média (mínimo-máximo)	225.000 (187.000-272.000)
RNI (média ± DP)	0,96±0,05
Diâmetro artéria doadora (média ± DP)	3,88±1,40
Diâmetro veia receptora (média ± DP)	3,57±1,36

DP = desvio padrão; IMC = índice de massa corporal; RNI = Razão Normalizada Internacional.

**Tabela 2:** Tipos de fístula arteriovenosa e número de cirurgias prévias.

Tipos de FAVs (%)	Radiocefálica	17 (36,17)
	Braquiobasílica	9 (19,15)
	Braquiobraquial	2 (4,25)
	Braquiocefálica	16 (34,04)
	Ulnar-basílica	1 (2,13)
	Ulno-ulnar	1 (2,13)
	Radiobasílica	1 (2,13)
Fístulas prévias (%)	0	66 (31)
	1	17 (8)
	2	15 (7)
	3	2 (1)

As taxas de perviedade primária e secundária foram, respectivamente, 89,4% e 93,6% no dia 1; 87,2% e 93,6% no dia 7; 86,96% e 96,62% no 30; e 82,98% e 91,49% no dia 60 do pós-operatório (Figura 2). Dos cinco pacientes que trombosaram no dia 1 do pós-operatório, dois foram submetidos a tromboembolectomia com cateter de Fogarty, seguido por reconfecção da anastomose e anticoagulação sistêmica com heparina não fracionada e varfarina; um paciente foi submetido a confecção de nova anastomose mais proximal; e dois pacientes não foram reabordados. Um paciente apresentou oclusão no 4º dia do pós-operatório e foi submetido a tromboembolectomia com cateter de Fogarty, seguido por reconfecção da anastomose e anticoagulação sistêmica com heparina não fracionada e varfarina. Esse paciente apresentou dois episódios de hematoma de ferida operatória, com necessidade de drenagem cirúrgica, sendo optado por suspensão da anticoagulação sistêmica. No 17º dia do pós-operatório, outro paciente apresentou trombose da FAV, necessitando de tromboembolectomia com cateter de Fogarty, seguido por reconfecção da anastomose e anticoagulação sistêmica com heparina não fracionada e varfarina. No 60º dia do pós-operatório, outro paciente apresentou oclusão da FAV após acidente de punção e não foi reabordado (Figura 1).

**Figura 2:** Perviedade primária e secundária das fístulas arteriovenosas para hemodiálise até os 60 dias de pós-operatório.

Ao comparar a VPS e o VF arterial e venoso no intraoperatório, observa-se um maior valor para as fístulas funcionantes tanto para perviedade primária quanto secundária comparadas às fístulas não funcionantes (Figura 3). Ao comparar a VPS e o VF arterial e venoso no intraoperatório entre os grupos que apresentaram FAV pérvia e não aos 60 dias, observa-se um maior valor para perviedade primária, mas não para secundária (Tabela 3).

**Figura 3:** Análise espectral da veia receptora em intraoperatório logo após a confecção da anastomose. 2A) Fístula não funcionante no 1º dia de pós-operatório. Velocidade sistólica de pico (VPS) 56 cm/s, volume de fluxo de 142 mL/min. 2B) Fístula funcionante no 1º dia de pós-operatório. VPS 192 cm/s, volume de fluxo de 463 mL/min.

**Tabela 3:** Comparação entre as velocidades de pico sistólico (VPS) e os volumes de fluxo (VF) arteriais e venosos ao ultrassom vascular com Doppler intraoperatório entre as FAV pérvias e não pérvias aos 30 e 60 dias pós-operatórios.

**Perviedade primária ao 30º dia pós-operatório. Valores apresentados em média e desvio padrão**			
	**Sim (n = 40)**	**Não (n = 7)**	**p**
VPS artéria cm/s	150 (70,08)	82,93 (33,66)	0,0082
VPS veia cm/s	170 (80,72)	93,66 (46,86)	0,0038
VF artéria mL/min	634 (444)	266 (261)	0,0084
VF veia mL/min	602 (462)	432 (272)	0,4451
			
**Perviedade secundária ao 30º dia pós-operatório. Valores apresentados em média e desvio padrão**			
	**Sim (n = 44)**	**Não (n = 3)**	**p**
VPS artéria cm/s	144,4 (69,47)	72,3 (37,26)	0,0498
VPS veia cm/s	165,5 (78,8)	58,47 (37,69)	0,0104
VF artéria mL/min	605,7 (439)	156,3 (100,1)	0,0132
VF veia mL/min	557,6 (330,8)	147,3 (78,14)	0,0028
			
**Perviedade primária ao 60º dia pós-operatório. Valores apresentados em média e desvio padrão**			
	**Sim (n = 39)**	**Não (n = 8)**	**p**
VPS artéria cm/s	150,2 (70,92)	88,79 (35,30)	0,0157
VPS veia cm/s	170,3 (81,76)	102, (49,49)	0,0065
VF artéria mL/min	629,5 (449,6)	333,3 (308,1)	0,042
VF veia mL/min	552,5 (323,7)	428,5 (397,9)	0,1185
			
**Perviedade secundária ao 60º dia pós-operatório. Valores apresentados em média e desvio padrão**			
	**Sim (n = 43)**	**Não (n = 4)**	**p**
VPS artéria cm/s	144,7 (70,26)	86,68 (41,86)	0,1058
VPS veia cm/s	165,6 (79,73)	84,10 (59,8)	0,0508
VF artéria mL/min	600,6 (443,8)	318,3 (334,0)	0,1411
VF veia mL/min	394,8 (498,9)	544,1 (322,3)	0,146

A partir desses resultados, foram construídos modelos de predição das perviedades primária e secundária no 30º dia após a confecção da FAV e da perviedade primária no 60º dia, de acordo com as variáveis hemodinâmicas. Entre as curvas ROC para perviedade primária no 30º dia do pós-operatório, o VF arterial mostrou-se como a variável com maior valor preditor para esse desfecho (*area under the curve* [AUC] = 0,8095) (Figura 4). Os pontos de corte com maior sensibilidade e especificidade para detectar FAVs funcionantes com perviedade primária no 30º dia do pós-operatório foram: 105,5 cm/s para VPS arterial, com sensibilidade de 72,5% (intervalo de confiança de 95% [IC95%]: 57,17-83,89%) e especificidade de 71,4% (IC95%: 35,89-94,92%); 106 cm/s para VPS venosa, com sensibilidade de 75% (IC95%: 59,81-85,81%) e especificidade de 71,4% (IC95%: 35,89-94,92%); e 290,5 mL/min para VF arterial, com sensibilidade de 80,6% (IC95%: 64,97-90,25%) e especificidade de 85,7% (IC95%: 48,69-99,27%) (Tabela 4).

**Figura 4:** Curvas *receiver operating characteristic* (ROC) comparando as variáveis ao ultrassom vascular com Doppler em intraoperatório e a perviedade primária aos 30 dias. VPS = velocidade sistólica de pico; VF = volume de fluxo; AUC = *area under the curve*.

**Tabela 4:** Sensibilidade e especificidade de diversos pontos de corte para velocidade de pico sistólico (VPS) e volume de fluxo (VF) arterial e venoso ao ultrassom vascular com Doppler intraoperatório para detectar perviedade primária e secundária aos 30 dias do pós-operatório e perviedade primária aos 60 dias do pós-operatório.

**Perviedade primária 30 dias**					
	**Valor**	**Sensibilidade**	**IC95%**	**Especificidade**	**IC95%**
VPS artéria cm/s	> 99,40	72,5	57,17% a 83,89%	57,1	25,05% a 84,18%
	> 105,5	72,5	57,17% a 83,89%	71,4	35,89% a 94,92%
	> 108,5	70,0	54,57% a 81,93%	71,4	35,89% a 94,92%
VPS veia cm/s	> 93,85	80,0	65,24% a 89,50%	57,1	25,05% a 84,18%
	> 99,15	77,5	62,50% a 87,68%	57,1	25,05% a 84,18%
	> 106,0	75,0	59,81% a 85,81%	71,4	35,89% a 94,92%
VF artéria mL/min	> 262,5	80,6	64,97% a 90,25%	71,4	35,89% a 94,92%
	> 290,5	80,6	64,97% a 90,25%	85,7	48,69% a 99,27%
	> 318,5	77,8	61,92% a 88,28%	85,7	48,69% a 99,27%
					
**Perviedade secundária 30 dias**					
	**Valor**	**Sensibilidade**	**IC95%**	**Especificidade**	**IC95%**
VPS artéria cm/s	> 93,85	77,3	63,01% a 87,16%	66,7	11,85% a 98,29%
	> 99,15	75,0	60,56% a 85,43%	66,7	11,85% a 98,29%
	> 106,0	72,7	58,15% a 83,65%	100,0	43,85% a 100,0%
VF veia mL/min	> 219,0	88,6	76,02% a 95,05%	66,7	11,85% a 98,29%
	> 225,0	86,4	73,29% a 93,60%	66,7	11,85% a 98,29%
	> 230,0	86,4	73,29% a 93,60%	100,0	43,85% a 100,0%
VF artéria cm/s	> 238,5	80,0	65,24% a 89,50%	66,7	11,85% a 98,29%
	> 251,5	77,5	62,50% a 87,68%	66,7	11,85% a 98,29%
	> 262,5	77,5	62,50% a 87,68%	100,0	43,85% a 100,0%
					
**Perviedade primária 60 dias**					
	**Valor**	**Sensibilidade**	**IC95%**	**Especificidade**	**IC95%**
VPS artéria cm/s	> 99,40	71,8	56,22% a 83,46%	50,0	21,52% a 78,48%
	> 105,5	71,8	56,22% a 83,46%	62,5	30,57% a 86,32%
	> 108,5	69,2	53,58% a 81,43%	62,5	30,57% a 86,32%
VPS veia cm/s	> 106,0	74,4	58,92% a 85,43%	62,5	30,57% a 86,32%
	> 111,1	71,8	56,22% a 83,46%	62,5	30,57% a 86,32%
	> 114,6	69,2	53,58% a 81,43%	62,5	30,57% a 86,32%
VF artéria mL/min	> 262,5	80,0	64,11% a 89,96%	62,5	30,57% a 86,32%
	> 290,5	80,0	64,11% a 89,96%	75,0	40,93% a 95,56%
	> 318,5	77,1	60,98% a 87,93%	75,0	40,93% a 95,56%

IC95% = intervalo de confiança de 95%.

Quanto à perviedade secundária 30 dias após a confecção da FAV, a variável com maior valor preditivo foi o VF venoso (AUC = 0,9545) (Figura 5). Para prever esse desfecho, os pontos de corte com maior sensibilidade e especificidade foram: 106 cm/s para VPS arterial, com sensibilidade de 72,7% (IC95%: 58,15-83,65%) e especificidade de 100% (IC95%: 43,85-100%); 230 mL/min para VF venoso, com sensibilidade de 86,4% (IC95%: 73,29-93,60%) e especificidade de 100% (IC95%: 43,85-100%); e 262,5 mL/min para VF arterial, com sensibilidade de 77,5% (IC95%: 62,5-87,68%) e especificidade de 100% (IC95%: 43,85-100%) (Tabela 4).

**Figura 5:** Curvas *receiver operating characteristic* (ROC) comparando as variáveis ao ultrassom vascular com Doppler em intraoperatório e a perviedade secundária aos 30 dias. VPS = velocidade sistólica de pico; VF = volume de fluxo; AUC = *area under the curve*.

Para a perviedade primária no 60º dia do pós-operatório, o VPS arterial, o VPS venoso e o VF arterial apresentaram AUC de 0,7692, 0,7356 e 0,7321, respectivamente (Figura 6). Os pontos de corte para os maiores valores de sensibilidade e especificidade para esse desfecho foram: 105,5 cm/s para VPS arterial, com sensibilidade de 71,8% (IC95%: 56,22-83,46%) e especificidade de 62,5% (IC95%: 30,57-86,32%), 106 cm/s para VPS venoso, com sensibilidade de 74,4% (IC95%: 58,92-85,43%) e especificidade de 62,5% (IC95%: 30,57-86,32%), e 290,5 mL/min para VF arterial, com sensibilidade de 80% (IC95%: 64,11-89,96%) e especificidade de 75% (IC95%: 40,93-95,56%).

**Figura 6:** Curvas *receiver operating characteristic* (ROC) comparando as variáveis ao ultrassom vascular com Doppler em intraoperatório e a perviedade primária aos 60 dias.

## DISCUSSÃO

Os parâmetros hemodinâmicos no intraoperatório mostraram-se potenciais preditores da perviedade das FAVs para hemodiálise. O VF arterial apresentou o maior valor preditor para a perviedade primária no 30º dia, com sensibilidade maior que 80% para o ponto de corte de 290,5 mL/min. Já para a perviedade secundária no 30º dia, o VF venoso foi a variável com maior valor preditor, com sensibilidade maior que 85% e especificidade de 100% para o ponto de corte de 230 mL/min. As VPS arterial e venosa demonstraram poder preditivo na perviedade primária do 60º dia, com pontos de corte de 105,5 cm/s e de 106 cm/s, respectivamente, e com sensibilidade maior que 70%.

Diversos estudos já demonstraram uma associação entre o VF intraoperatório e os principais desfechos das FAVs. Saucy et al.^13^ avaliaram preditores para a falha na maturação das FAVs radiocefálicas nos primeiros 30 dias após sua confecção, encontrando o VF intraoperatório de 120 mL/min como ponto de corte, mas com menor taxa de perviedade (77,58%). Cyrek et al.^14^ compararam FAVs radiocefálicas de alto (> 200 mL/min) e baixo VF (< 200 mL/min) no intraoperatório e observaram taxas de perviedade primária e secundária significativamente mais altas nas FAVs de alto fluxo (100% e 93,15%, respectivamente) quando comparadas às de baixo fluxo (81,25% e 75%, respectivamente) em 1 ano. Outros estudos observacionais reiteram essa mesma relação entre o VF pós-operatório e a falha da FAV, encontrando pontos de corte que variam entre 160 mL/min e 300 mL/min^15^^,^^16^.

Em relação aos valores de VPS, diversos estudos corroboram com a associação encontrada. Karanan et al.^17^ analisaram o desfecho das FAVs de acordo com o valor da VPS arterial nos dias 1 e 8 do pós-operatório, confirmando a VPS como preditor do desfecho da FAV de forma significativa. Em concomitância, Abreu et al.^18^ conduziram um estudo com acompanhamento de 5 anos que demonstrou VPS em artérias ulnar e radial como valor preditor para perviedade secundária.

Observamos algumas limitações neste estudo. Tratou-se de estudo em centro único, com único observador, não sendo validadas ou avaliadas as variações e vieses interobservadores. A amostra foi bastante heterogênea, com uma grande variedade de tipos de FAVs. Foram utilizados dois tipos diferentes de aparelhos de ultrassonografia. O aparelho Philips CX 50 foi utilizado para o exame intraoperatório, e o Toshiba Aplio 300, para os exames pós-operatórios. Pode haver uma pequena variação nos valores de VPS e VF entre os aparelhos. Neste estudo, apresentou-se um reduzido número de pacientes; dessa forma, optou-se pela análise univariada, a qual pode apresentar algumas limitações, sobretudo quanto à possibilidade de predição de determinado desfecho. Outra limitação importante foi pequeno número de eventos de não perviedade primária no dia 30, o que pode ter contribuído para ICs muito amplos.

O tema abordado nesta pesquisa permanece em aberto e ainda pouco estudado. Por se tratar de propedêutica não invasiva, de ampla distribuição e acesso, com ausência de efeitos colaterais e de riscos para o paciente, a ultrassonografia vascular com Doppler realizada em intraoperatório é uma importante ferramenta para o cirurgião vascular. Esse exame fornece variáveis importantes e em tempo real, as quais podem se correlacionar com o sucesso a curto prazo da cirurgia e modificar a estratégia cirúrgica no intraoperatório – como uma reconfecção do acesso ou até mesmo mudança de local da anastomose – a depender dos valores de VPS e VF. A VPS, apesar de apresentar menor sensibilidade e especificidade quando comparada ao VF, é uma variável de fácil execução e análise, disponível em quase todos os aparelhos de ultrassonografia vascular.

## CONCLUSÕES

A VPS e o VF ao ultrassom vascular com Doppler intraoperatório são preditores de perviedade precoce na cirurgia de FAV para hemodiálise. Ambos representam parâmetros de avaliação confiável para predição da falência precoce da FAV e oferecem a possibilidade de mudança da estratégia cirúrgica no intraoperatório para atingir melhores desfechos no pós-operatório.
